# Impact of electrocardiographic morphology on clinical outcomes in patients with non-ST elevation myocardial infarction receiving coronary angiography and intervention: a retrospective study

**DOI:** 10.7717/peerj.8796

**Published:** 2020-05-07

**Authors:** Chiung-Jen Wu, Kuo-Ho Yeh, Hui-Ting Wang, Wen-Hao Liu, Huang-Chung Chen, Han-Tan Chai, Wen-Jung Chung, Shukai Hsueh, Chien-Jen Chen, Hsiu-Yu Fang, Yung-Lung Chen

**Affiliations:** 1 Division of Cardiology, Department of Internal Medicine, Kaohsiung Chang Gung Memorial Hospital, Kaohsiung, Taiwan; 2 Emergency Department, Kaohsiung Chang Gung Memorial Hospital, Kaohsiung, Taiwan

**Keywords:** Non-ST segment elevation myocardial infarction, Electrocardiographic morphology, ST depression, Percutaneous coronary intervention, Clinical outcomes

## Abstract

**Background:**

The impact of electrocardiography (ECG) morphology on clinical outcomes in patients with non-ST segment elevation myocardial infarction (NSTEMI) receiving percutaneous coronary intervention (PCI) is unknown. This study investigated whether different ST morphologies had different clinical outcomes in patients with NSTEMI receiving PCI.

**Methods:**

This retrospective study analyzed record-linked data of 362 patients who had received PCI for NSTEMI between January 2008 and December 2010. ECG revealed ST depression in 67 patients, inverted T wave in 91 patients, and no significant ST-T changes in 204 patients. The primary endpoint was long-term all-cause mortality. The secondary endpoint was long-term cardiac death and non-fatal major adverse cardiac events.

**Results:**

Compared to those patients whose ECG showed an inverted T wave and non-specific ST-T changes, patients whose ECG showed ST depression had more diabetes mellitus, advanced chronic kidney disease (CKD) and left main artery disease, as well as more in-hospital mortality, cardiac death and pulmonary edema during hospitalization. Patients with ST depression had a significantly higher rate of long-term total mortality and cardiac death. Finally, multiple stepwise Cox regression analysis showed that an advanced Killip score, age, advanced CKD, prior percutaneous transluminal coronary angioplasty and ST depression were independent predictors of the primary endpoint.

**Conclusions:**

Among NSTEMI patients undergoing coronary angiography, those with ST depression had more in-hospital mortality and cardiac death. Long-term follow-up of patients with ST depression consistently reveals poor outcomes.

## Introduction

According to clinical guidelines, the definition of non-ST segment elevation myocardial infarction (NSTEMI) is clinical chest tightness and elevation of cardiac enzyme without ST elevation on electrocardiography (ECG) (Nieto et al., 2007). Early percutaneous coronary intervention (PCI) is one of the standard treatments for high-risk NSTEMI patients, including those patients with refractory angina, unstable hemodynamic status, heart failure symptoms, malignant tachyarrhythmia, or a higher risk score (Nieto et al., 2007; Cannon et al., 2001; Fox et al., 2005; Wallentin et al., 1999). The ECG pattern is one of the components of some risk scores used for risk stratification, especially in the mainly pre-thrombolytic and thrombolytic era (Nieto et al., 2007). However, the influence of different ECG patterns on the clinical outcome of NSTEMI is unclear, especially in the PCI era, and the impact of the ECG pattern on risk stratification may be different in the thrombolytic and PCI era. Furthermore, no studies have focused on the impact of ECG patterns on long-term outcomes in this specific population after PCI. This study was aimed at evaluating the prognostic impact of clinical ECG patterns on clinical outcomes of NSTEMI patients undergoing PCI.

## Materials & Methods

### Setting and patient population

This study retrospectively analyzed all patients who had received PCI after being diagnosed with NSTEMI at Kaohsiung Chang Gung Memorial Hospital from January 2008 to December 2010. Data were collected as previously described in Wang, Chen & Wu (2016). Specifically, the definition of NSTEMI was typical chest pain, troponin-I elevation ≥ 0.5 ng/ml and no ST-segment elevation by clinical ECG finding. The inclusion criterion in our study was patients with NSTEMI who received angiography within 72 h after presentation. The exclusion criteria included the availability of only one set of troponin-I data, the absence of a rising pattern of the troponin-I level during follow-up, or delayed PCI (more than 72 h) due to other etiologies (mostly to poor renal function, active bleeding and sepsis). Stent implantations for stenotic vessels were performed according to clinical guidelines  (Amsterdam et al., 2014). Data collection included baseline characteristics, ECG findings, left ventricular ejection fraction (LVEF) obtained by echocardiography or left ventricular angiography, coronary angiography and PCI data. Blood samples were evaluated when patients arrived at the ER (emergency room), and ECG was performed after the patients arrived, or if they had acute typical chest pain during hospitalization. We analyzed the in-hospital outcomes, including total mortality, cardiac death, ventricular arrhythmia and pulmonary edema. Patients received continued clinical follow-up after being discharged. The primary endpoint of this study was long-term mortality. The secondary endpoint was long-term cardiac death and non-fatal major adverse cardiac events (MACE), which were defined as target vessel revascularization, myocardial infarction (MI), stroke, and hospitalization for heart failure. An advanced Killip score was defined as a score ≥ 3 and advanced chronic kidney disease (CKD) was defined as an estimated glomerular filtrating rate <60 ml/min/1.73 m2. The study protocol was approved by the Institutional Review Committee for Human Research of Kaohsiung Chang Gung Memorial Hospital (201900344B0). The need for consent was waived by the Institutional Review Board of Kaohsiung Chang Gung Memorial Hospital.

### Electrocardiography (ECG) definition

The ECG findings were classified as no significant ST-T change, ST depression or T wave inversion. ST depression was defined as horizontal or down-sloping ST segment depression ≥1 mV at 80 ms after a J point over at least 2 consecutive leads. T wave inversion was defined as the amplitude of an inverted T wave of at least 1 mm over at least 2 consecutive leads. Patients with 1 mm ST-segment depression and T-wave inversion were classified as having ST depression. ECG results that did not meet these criteria without significant ST segment elevation were defined as showing no significant ST-T change. Those patients who had some ST elevation, but not enough to meet STEMI criteria, were also classified as “no significant ST-T change”.

### Coronary angiography and stenting

Coronary stent implantation was performed using procedures and protocols described in our previous publications (Wang, Chen & Wu, 2016; Chen et al., 2007; Chen et al., 2011). Briefly, diagnostic catheterization and intervention were performed as soon as possible within 72 h after the diagnosis of NSTEMI was confirmed. Before the procedure, dual antiplatelets (aspirin and clopidogrel) were given, and unfractionated heparin or low molecular weight heparin was given for heparinization, based on renal function. We did total revascularization for culprit lesions and all other radiographically significant stenoses at the index procedure for those patients presenting with NSTEMI, or treated non-culprit lesions at the staged procedure during the index admission. Left main (LM) lesions with ≥50% stenosis and lesions of the main trunk of triple vessels with more than 70% stenosis were usually treated (stenting if the vascular reference was ≥ 2.5 mm or balloon angioplasty for vascular references of 2.0–2.5 mm). Chronic total occlusive lesions usually did not undergo revascularization during the index procedure. We usually evaluated myocardial perfusion using a thallium-201 scan thereafter, during the OPD follow-up, and decided if revascularization was indicated for those patients. Most lesions were predilated with a 1:1 ratio of balloons prior to stent implantation. Most patients underwent post-stenting dilatation with a high-pressure balloon. Multiple-vessel disease (MVD) was defined as stenosis ≥50% in ≥2 major epicardial coronary arteries.

### Post-percutaneous coronary intervention management

Patients were treated according to current clinical guidelines for post-PCI management (Wang, Chen & Wu, 2016). Our patients were covered under Taiwan’s National Health Insurance (NHI) program, which is a compulsory single-payer healthcare system featuring care coverage for more than 99.8% of the population in Taiwan. In accordance with NHI reimbursement criteria, dual antiplatelet therapies were given for at least 9 months and statin therapy was given for those patients with low-density lipoprotein ≥ 100 mg/dl, in the absence of contraindications. Patients were referred for cardiac rehabilitation routinely and the compliance rate was about 90%. Major risk factors such as smoking, hypertension, dyslipidemia, physical inactivity, obesity, and diabetes mellitus (DM) were also treated.

### Statistical analysis

Unless otherwise stated, data were expressed as means ± standard deviation or as a number (percentage). Differences in continuous variables were analyzed using one-way analysis of variance for continuous variables, and categorical variables were analyzed using the chi-square test. Pairwise *post hoc* multiple comparisons between any 2 study groups were made using the Bonferroni adjustment. For long-term outcome analysis, those patients with T wave inversion and no significant ST change pattern were defined as the no-ST depression group. The independent predictors of long-term mortality were analyzed using the multiple stepwise Cox regression method. Event-free survival in patients with and without ST depression and also with different ECG patterns was evaluated and compared by the Kaplan–Meier method and log rank test. Statistical analysis was performed with statistical software (SPSS Statistics for Windows, version 17.0; SPSS Inc., Chicago, IL, USA). A 2-tailed *p* value <0.05 was considered statistically significant.

## Results

### Baseline characteristics and in-hospital outcomes of the study patients

During the study period, 409 patients who had a clinical diagnosis of NSTEMI received coronary angiography and PCI. Forty-seven patients who met our exclusion criteria were excluded from analysis. Of the 362 patients enrolled in this study, 264 (72.9%) were males and the average age was 65.6 ± 12.2 years. Sixty-seven (18.5%) patients were classified as having ST depression, 91 (25.1%) as having T wave inversion, and the remaining 204 (56.4%) as having no significant ST-T change pattern. Patients with ST depression had a significantly higher percentage of DM and CKD than patients with T wave inversion and no significant ST-T changes (both *P* < .05). More of the patients with ST depression and T wave inversion had a past history of old MI and coronary artery bypass surgery (CABG) than the patients with no significant ST-T changes (both *P* <0.05). More patients with ST depression had an advanced Killip score, and patients with T wave inversion had a lower body mass index than patients with no significant ST-T change (both *P* <0.05). There was no significant difference in age, male gender, current smoker, hypertension, dyslipidemia, old stroke, peripheral vascular disease, percutaneous transluminal coronary angioplasty, or time to angiography among patients with different ECG patterns. Patients with ST depression had a higher creatinine level than other patients and a higher troponin-I level than patients with T wave inversion (both *P* <0.05). There was no significant difference in LVEF among patients with different ECG patterns (Table 1).

**Table 1:** Baseline characteristics, angiographic findings and clinical outcomes of 362 study patients according to the presenting ECG.

Variables	No ST changes (*n* = 204)	Inverted T wave (*n* = 91)	ST depression (*n* = 67)	*P* Value
Age (yrs)	64.7 ± 12.2	65.2 ± 13.4	68.8 ± 10.3	0.059
Male gender	77.5% (158)	68.1% (62)	65.7% (44)	0.084
Current smoker	24.0% (49)	28.6% (26)	17.9% (12)	0.301
Hypertension	74.5% (152)	70.3% (64)	76.1% (51)	0.669
Diabetes mellitus	46.1% (94)^a^	35.2% (32)^a^	52.2% (35)^b^	0.080
Dyslipidemia	38.7% (79)	36.3% (33)	37.3% (25)	0.918
Old stroke	16.2% (33)	22.0% (20)	19.4% (13)	0.473
Old MI	8.8% (18)^a^	17.6% (16)^b^	17.9% (12)^b^	0.042
Advanced CKD^*^	40.2% (82)^a^	44.0% (40)^a^	65.7% (44)^b^	<0.001
PVD	3.4% (7)	4.4% (4)	7.5% (5)	0.379
Prior PTCA	15.2% (31)	13.2% (12)	23.9% (16)	0.161
Prior CABG	0% (0)^a^	3.3% (3)^b^	6.0% (4)^b^	0.005
BMI (kg/m2)	25.4 ± 3.6^a^	24.1 ± 3.4^b^	25.2 ± 3.6^ab^	0.020
Advanced Killip score^†^	12.7% (26)^a^	19.8% (18)^ab^	32.8% (22)^b^	0.001
LVEF (%)	60.7 ± 12.3	59.3 ± 14.6	56.2 ± 15.3	0.070
Creatinine level (mg/dl)	1.33 ± 1.07^a^	1.26 ± 0.85^a^	1.87 ± 1.70^b^	0.002
eGFR (ml/min/1.73m^2^)	68.1 ± 28.7^a^	66.6 ± 28.5^a^	51.2 ± 25.3^b^	<0.001
Troponin-I (ng/ml)	9.88 ± 24.6^ab^	7.4 ± 9.8^b^	19.4 ± 50.0^a^	0.025
CAG/PTCA finding				
Time to angiography				0.654
<24 h	38.7% (79)	45.1% (41)	37.3% (25)	
24–48 h	12.7% (26)	12.1% (11)	17.9% (12)	
48–72 h	48.5% (99)	42.9% (39)	44.8% (30)	
Significant CAD	89.2% (182)	84.6% (77)	82.1% (55)	0.259
Multiple-vessel disease	67.2% (137)	62.6% (57)	71.6% (48)	0.489
LM	9.8% (20)^a^	12.1% (11)^a^	25.4% (17)^b^	0.005
Total stent number	1.6 ± 1.1	1.5 ± 1.1	1.8 ± 1.4	0.255
Stent type				0.277
Drug eluting stent	45.6% (93)	48.4% (44)	34.3% (23)	
Bare metal stent	43.6% (89)	37.4% (34)	47.8% (32)	
Final TIMI-3 flow	98.0% (200)	98.9% (90)	100.0% (67)	0.474
Complete revascularization	93.1% (190)	93.4% (85)	89.6% (60)	0.585
Medication				
Aspirin	81.9% (167)^a^	79.1% (72)^a^	70.1% (47)^b^	0.124
Clopidogrel	89.7% (183)^a^	81.3% (74)^b^	85.1% (57)^ab^	0.132
ACEI/ARB	70.1% (143)	64.8% (59)	61.2% (41)	0.350
Beta-blocker	63.7% (130)	68.1% (62)	56.7% (38)	0.337
Statin	56.9% (116)	54.9% (50)	56.7% (38)	0.952
In-hospital events				
Total mortality	2.9% (6)^a^	1.1% (1)^a^	9.0% (6)^b^	0.024
Cardiac death	2.5 (5)^a^	0% (0)^a^	7.5% (5)^b^	0.017
VT/VF	2.9% (6)	2.2% (2)	4.5% (3)	0.706
PE	17.2% (35)^a^	18.7% (17)^a^	46.3% (31)^b^	<0.001
Long-term outcome				
Follow-up duration (months)	69.7 ± 43.9^a^	72.1 ± 43.8^a^	48.0 ± 41.2^b^	0.001
Total mortality	15.2% (31)^a^	8.8% (8)^a^	31.3% (21)^b^	0.001
Cardiac death	7.4% (15)^a^	0.0% (0)^b^	14.9% (10)^c^	0.001
MACE^‡^	31.9% (65)	35.2% (32)	32.8% (22)	0.856
TVR	14.2% (29)	12.1% (11)	13.4% (9)	0.885
MI	9.3% (19)	13.2% (12)	9.0% (6)	0.556
Stroke	4.9% (10)	5.5% (5)	4.5% (3)	0.956
Heart failure	9.8% (20)	9.9% (9)	13.4% (9)	0.686

**Notes.**

Data are expressed as means ± SD or % (n).

ACEI/ARB angiotensin-converting enzyme inhibitors/angiotensin-receptor blockers BMI body mass index CABG coronary artery bypass surgery CAG coronary angiography ECG electrocardiogram eGFR estimated glomerular filtration rate ESRD end stage renal disease HF heart failure LVEF left ventricular ejection fraction MACE major adverse cardiac event MI myocardial infarction PCI percutaneous cardiac intervention PE pulmonary edema PTCA percutaneous transluminal coronary angioplasty PVD peripheral vascular disease TIMI thrombolysis in myocardial infarction TVR target vessel revascularization VF ventricular fibrillation VT ventricular tachycardia

* eGFR < 60 ml/min/1.73 m^2^.

† Killip score ≥ 3.

‡ Target vessel revascularization, myocardial infarction, stroke and hospitalization for heart failure.

Different letters (a, b, c, i.e.,^a^ vs. ^b^, vs.^c^) indicate significant difference between groups (*P* < 0.05).

Total revascularization for culprit lesions and all other radiographically significant stenoses were achieved in 95.6% of patients during hospitalization (93.8% at the index procedure simultaneously for those patients presenting with NSTEMI and 1.8% at the staged procedure for non-culprit lesions during the index admission.) In terms of coronary angiography and PCI findings, more patients presenting with ST depression had left main disease than patients presenting with other ECG findings (*P* <0.05). There was no difference in the percentage of significant coronary artery disease (CAD), MVD, total stents number and types and post-PCI final thrombolysis in MI (TIMI)-3 flow among patients with different ECG patterns. Patients presenting with an ST depression pattern had more total in-hospital mortality, cardiac death and pulmonary edema events (*P* <0.05). The number of events of in-hospital ventricular tachycardia and fibrillation did not differ.

### Primary outcome analysis: long-term mortality

The mean follow-up period was 66.3 ± 44.1 months: 60 (16.6%) patients died during that period. Total mortality was significantly higher among patients with ST depression than among those with T wave inversion and no significant ST-T change (*P* = 0.001) (Table 1).

Compared to the patients who survived, those who had died were significantly older (71.4 ± 9.4 vs. 64.4 ± 12.4 years, *p* < 0.001), and more likely to have DM, prior percutaneous transluminal coronary angioplasty (PTCA), advanced CKD, advanced Killip score and lower LVEF (all *p* < .05). Those patients who died were also more likely to have an ECG pattern of ST depression than the patients who survived (35.0 vs. 15.2%, *p* < 0.001) (Table 2). All variables with a *P* value ≤ 0.1 by univariate analysis were put into multiple stepwise Cox regression analysis, which showed that advanced Killip score (hazard ratio [HR] = 3.417, 95% confidence interval (CI): 1.966–5.939, *P* <0.001), age (HR = 1.043, 95% CI [1.015–1.072], *P* = 0.002), prior PTCA (HR = 1.987, 95% CI [1.110–3.558], *P* = 0.021), advanced CKD (HR = 1.871, 95% CI [1.044–3.354], *P* = 0.035) and ECG pattern of ST depression (HR = 1.809, 95% CI [1.015–3.223], *P* = 0.044) were the only independent predictors of long-term mortality (Table 3).

**Figure 1:**
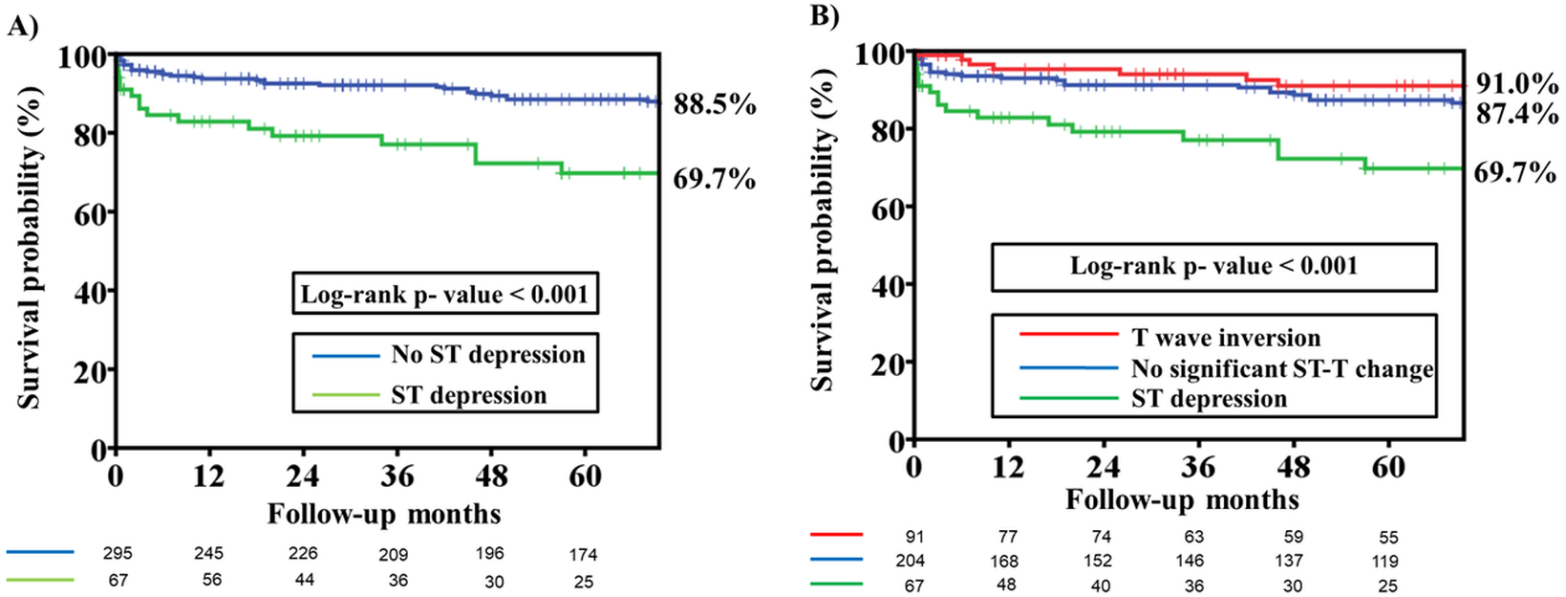
Kaplan–Meier estimates of total mortality among patients with different ST segment morphology on the presenting electrocardiography. (A) Kaplan–Meier estimates of total mortality among patients with and without ST depression on the presenting electrocardiography. The analysis reveals that the overall survival rate at 66 months was significantly lower in the ST depression group than in the no-ST depression group (69.7% versus 88.5%, Log-rank *P* value < 0.001). (B) Kaplan–Meier estimates of total mortality among patients with no significant ST-T change, ST depression and T wave inversion on presenting electrocardiography. The analysis reveals that the overall survival rate at 66 months was significantly lower in the ST depression group than in the no significant ST-T change and T wave inversion group (69.7% versus 87.4% versus 91.0%, Log-rank *P* value < 0.001).

Figure 1A compares the Kaplan–Meier curves for primary endpoint among patients with and patients without an ECG pattern of ST depression. Long-term mortality was significantly higher in patients with ST depression than in those without ST depression (Log-rank *p* value <0.001). Figure 1B compares the Kaplan–Meier curves for primary endpoint among patients with different ECG patterns. Patients with ST depression were found to have a higher rate of long-term mortality than patients with T wave inversion and no significant ST-T changes (Log-rank *p* value <0.001).

### Secondary endpoint analysis: long-term cardiac death and non-fatal MACE

**Figure 2:**
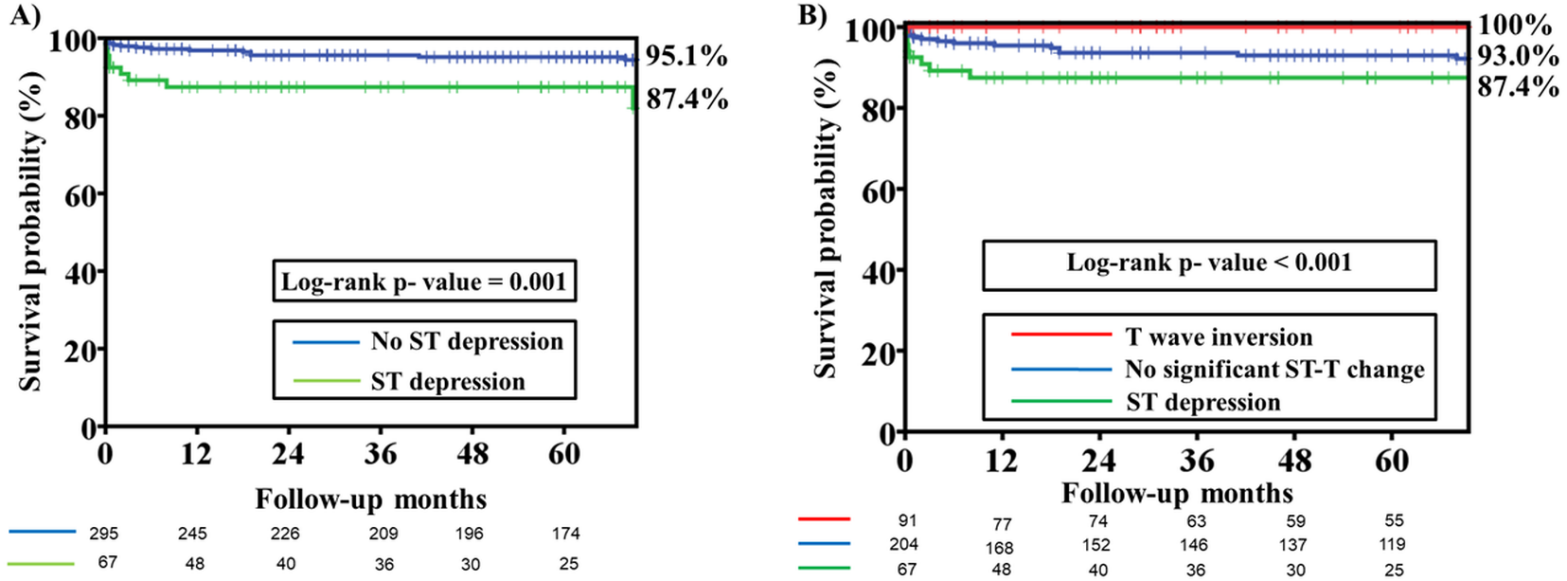
Kaplan–Meier estimates of cardiac death among patients with different ST segment morphology on the presenting electrocardiography. (A) Kaplan–Meier estimates of cardiac death among patients with and without ST depression on the presenting electrocardiography. The analysis reveals that the freedom from cardiac death rate at 66 months was significantly lower in the ST depression group than in the no-ST depression group (87.4% versus 95.1%, Log-rank *P* value =0.001). (B) Kaplan–Meier estimates of cardiac death among patients with no significant ST-T change, ST depression and T wave inversion on the presenting electrocardiography. The analysis reveals that the freedom from cardiac death rate at 66 months was significantly lower in the ST depression group than in the no significant ST-T change and T wave inversion group (87.4% versus 93.0% versus 100%, Log-rank *P* value < 0.001).

**Figure 3:**
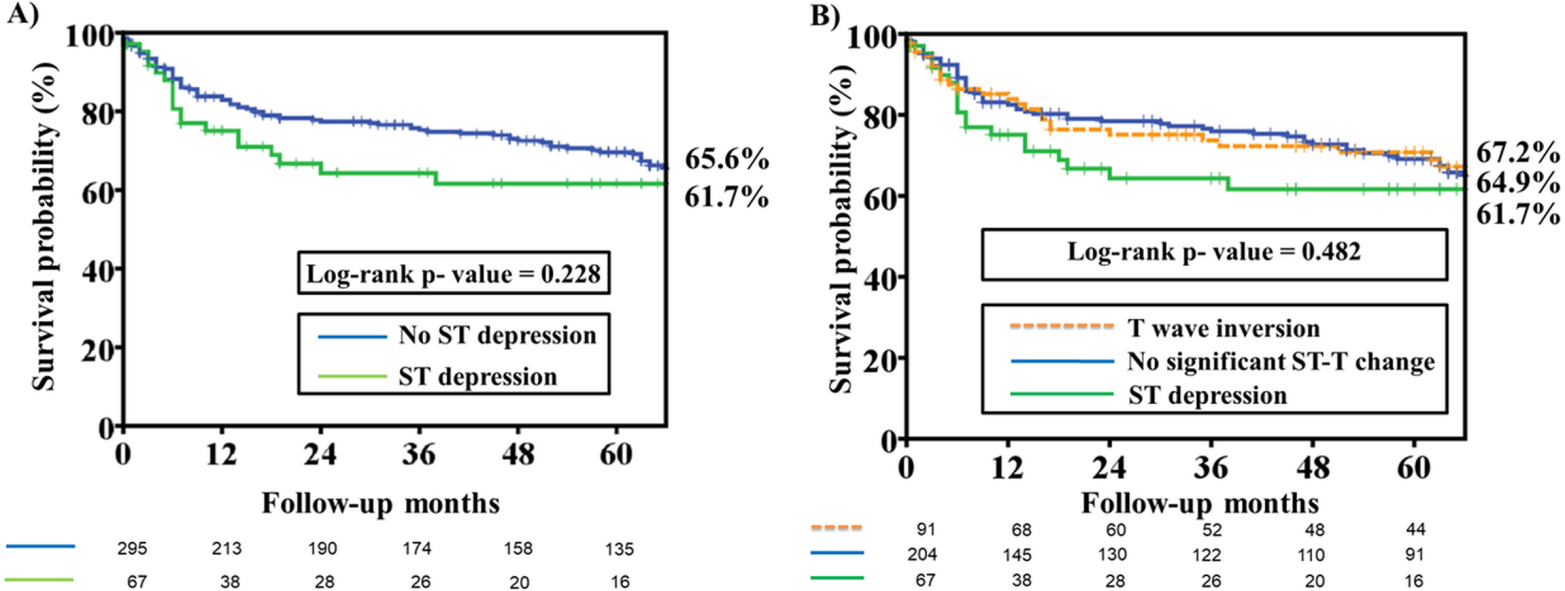
Kaplan–Meier estimates of non-fatal major adverse cardiac events (MACE) among patients with different ST segment morphology on the presenting electrocardiography. (A) Kaplan–Meier estimates of non-fatal MACE, which were defined as target vessel revascularization, myocardial infarction, stroke, and hospitalization for heart failure, among patients with and without ST depression on the presenting electrocardiography. The analysis illustrates that there was no difference in the freedom from non-fatal MACE rate at 66 months among patients with ST depression and no-ST depression ECG patterns (61.7% versus 65.6%, Log-rank *P* value =0.228). (B) Kaplan–Meier estimates of non-fatal MACE among patients with no significant ST-T change, ST depression and T wave inversion on the presenting electrocardiography. The analysis reveals that there were no differences in non-fatal MACE among patients with ST depression, no significant ST-T changes and T wave inversion (61.7% versus 64.9% versus 67.2%, Log-rank *P* value = 0.482).

Long-term cardiac death was significantly higher in patients with an ST depression ECG pattern than in patients with T wave inversion and no significant ST change pattern (*P* = 0.001) (Table 1). There was no difference in long-term non-fatal MACE among patients with different ECG patterns. The cardiac death rate was significantly higher in patients with an ST depression ECG pattern than in those without ST depression (Fig. 2A; Log-rank *p* value = 0.001). In Fig. 2B, a comparison of Kaplan–Meier curves for cardiac death among patients with different ECG patterns revealed that patients with ST depression had a higher rate of long-term cardiac death than patients with T wave inversion and no significant ST-T changes (Log-rank *p* value <0.001). Kaplan–Meier curves for non-fatal MACE among patients with and without an ST depression ECG patterns revealed that there was no difference in both groups (Log-rank *P* value = 0.228) (Fig. 3A). There was no difference in non-fatal MACE among patients with ST depression, no significant ST-T changes and T wave inversion (Log-rank *P* value = 0.482) (Fig. 3B).

## Discussion

There are several important findings in this study. First, NSTEMI patients presenting with an ST depression ECG pattern had a higher percentage of DM, advanced CKD and LM disease than those patients presenting with other ECG patterns. Second, the NSTEMI patients presenting with an ST depression ECG pattern had not only higher rates of in-hospital total mortality, cardiac death and pulmonary edema but also higher rates of total mortality and cardiac death during long-term follow-up. Third, an advanced Killip score, older age, advanced CKD, prior PTCA and an ST depression ECG pattern were independent predictors of long-term total mortality.

**Table 2:** Baseline characteristics, angiographic findings and clinical outcomes of 362 study patients with and without long term mortality.

Variables	Mortality (*n* = 60)	Alive (*n* = 302)	*P* Value
Age (yrs)	71.4 ± 9.4	64.4 ± 12.4	<0.001
Male gender	65.0% (39)	74.5% (225)	0.130
Current smoker	16.7% (10)	25.5% (77)	0.185
Hypertension	78.3% (47)	72.8% (220)	0.425
Diabetes mellitus	60.0% (36)	41.4% (125)	0.010
Dyslipidemia	38.3% (23)	37.7% (114)	0.932
Old stroke	23.3% (14)	17.2% (52)	0.263
Old myocardial infarction	13.3% (8)	12.6% (38)	0.873
Prior PTCA	28.3% (17)	13.9% (42)	0.006
Prior CABG	1.7% (1)	2.0% (6)	1.000
Peripheral vascular disease	8.3% (5)	3.6% (11)	0.106
Advanced CKD^a^	66.7% (40)	41.7% (126)	0.001
Body mass index (kg/m^2^)	25.1 ± 3.8	25.0 ± 3.5	0.932
Advance Killip score^b^	43.3% (26)	13.2% (40)	<0.001
Troponin-I	19.6 ± 52.5	9.3 ± 21.2	0.137
LVEF	54.5 ± 13.9	60.5 ± 13.5	0.003
Time to angiography			0.177
<24 h	30.0% (18)	42.1% (127)	
24–48 h	18.3% (11)	12.6% (38)	
48–72 h	51.7% (31)	45.4% (137)	
ECG changes			<0.001
ST depression	35.0% (21)	15.2% (46)	
No ST depression	65.0% (39)	84.8% (256)	
Multiple-vessel disease	66.7% (40)	66.9% (202)	0.974
Left main disease	20.0% (12)	11.9% (36)	0.092
Total stent number	1.4 ± 1.0	1.3 ± 0.7	0.326
Stent type			0.407
Drug eluting stent	36.7% (22)	45.7% (138)	
Bare metal stent	50.0% (30)	41.4% (125)	
Post-PCI TIMI-3 flow	96.7% (58)	99.0% (299)	0.156
Complete revascularization	91.7% (55)	92.7% (280)	0.778

**Notes.**

Data are expressed as means ± SD or % (n).

CABG coronary artery bypass surgery CKD chronic kidney disease ECG electrocardiogram ESRD end stage renal disease LVEF Left Ventricular Ejection fraction PCI percutaneous cardiac intervention PTCA percutaneous transluminal coronary angioplasty TIMI Thrombolysis in Myocardial Infarction

a Estimated glomerular filtration rate < 60 ml/min/1.73 m^2^.

b Killip score ≥ 3.

**Table 3:** Univariate and multivariate analysis of predictors for long-term mortality.

Variables	Univariate analysis		Multivariate analysis	
	HR (95% CI)	*P* value	HR (95% CI)	*P* value
Advanced Killip score^a^	5.436 (3.232–9.143)	<0.001	3.417 (1.966–5.939)	<0.001
Age (year)	1.065 (1.039–1.093)	<0.001	1.043 (1.015–1.072)	0.002
Prior PTCA	2.443 (1.391–4.293)	0.002	1.987 (1.110–3.558)	0.021
Advanced CKD^b^	2.782 (1.670–4.636)	<0.001	1.871 (1.044–3.354)	0.035
ST depression	3.113 (1.826–5.308)	<0.001	1.809 (1.015–3.223)	0.044
LM disease	2.516 (1.326–4.775)	0.005	1.919 (1.069–3.445)	0.094
DM	2.067 (1.232–3.467)	0.006	1.083 (0.611–1.922)	0.784
LVEF (%)	0.967 (0.950–0.985)	<0.001	1.002 (0.981–1.023)	0.868

**Notes.**

CI confidence interval CKD chronic kidney disease DM diabetes mellitus HR hazard ratio LM left main LVEF left ventricular ejection fraction PTCA percutaneous transluminal coronary angioplasty

a Killip score ≥ 3.

b Estimated glomerular filtration rate < 60 ml/min/1.73 m^2^.

An ST depression ECG pattern is one of the components of the TIMI risk score commonly used to predict 14-day outcomes of unstable angina and NSTEMI in clinical practice (Antman et al., 2000). It is surprising that only a few studies have compared intermediate or long-term (using only 6–60 months of mean follow-up time) outcomes of acute coronary syndrome (ACS)/NSTEMI between patients with and without ST depression in the mainly pre-thrombolytic and thrombolytic era (Cannon et al., 1997; Hyde et al., 1999; Eagle et al., 2004; Jacobs Jr et al., 1999). To the best of our knowledge, this is the only study to compare the long-term outcome of NSTEMI patients with and without ST depression in the intervention era. The TIMI III Registry showed that in patients with non-ST elevation ACS, an ST deviation of as little as 0.05 mV increased the risk of death or MI by approximately 2-fold, both at 30 days and at 1 year (Cannon et al., 1997). Hyde et al. (1999) reported that ST depression of 0.05 mV or more on the admission ECG was related to 4-year mortality rates; the risk of death increased as ST depression increased. In contrast, T-wave inversion of 0.1 mV or more was associated with only a modest increase or no increase at all in the subsequent risk of death or MI in the same study (Hyde et al., 1999). In that study, 67% of patients received intravenous heparin, 37% underwent angiography, and 35% underwent revascularization. Researchers with the GRACE study (Global Registry of Acute Coronary Events) developed a score that allows us to predict patient mortality 6 months after being discharged following an ACS episode; an ST decrease ≥ 1 mm was one of 9 prognostic variables used to determine the patient’s probability of death (Eagle et al., 2004). It is worth noting that 33% of patients received PCI or CABG in this study, and having no in-hospital PCI was another predictor of 6-month mortality. The PREDICT score is the only score developed to predict a relatively long-term prognosis (6 years) after hospitalization for ACS in the mainly pre-thrombolytic and thrombolytic era (Jacobs Jr et al., 1999). Only 11% of patients received thrombolytic therapy in the study. ST segment depression >1 mm horizontal or downward sloping was a major predictor of long-term mortality in this score. Our study was conducted in the PCI era and all patients received angiography; 87% of these patients received PCI due to significant CAD. ST depression is still an independent predictor of in-hospital and long-term mortality. Our study confirms the previous finding that only those patients with an ST depression pattern, but not with T wave inversion, had a worse in-hospital and long-term mortality (more than 5 years) outcome than patients with no significant ST-T changes. An ST depression ECG pattern may be a very easy and important hint for clinical physicians to use to identify those NSTEMI patients who are at higher risk of in-hospital and long-term mortality. Patients with ST depression may need more aggressive therapy and management, including risk modification and even immediate or early intervention (for those patients with refractory angina, unstable hemodynamic status, heart failure symptoms, malignant tachyarrhythmia, or a higher TIMI or GRACE risk score, according to clinical guidelines). Further studies focusing on the role of an ST depression ECG pattern in risk stratification and the impact of immediate or early intervention on outcomes are needed for further evaluation.

A previous study showed a T-wave inversion is sensitive for ischemia but is less specific, unless it is marked (≥0.3 mV) (Hyde et al., 1999). This may explain why T-wave inversion could not independently predict total mortality in previous studies and in our study.

Furthermore, our study showed that the percentages of DM, advanced CKD and LM disease were higher in patients with ST depression than in patients with other ECG patterns. A consistent finding was that the risk of adverse outcomes increased with diabetes, impaired renal function and prior CAD during ACS (Wang, Chen & Wu, 2016; Jacobs Jr et al., 1999; Cohen, 2016; Fox et al., 2006; Roe et al., 2011; Fox et al., 2014; Mueller et al., 2004). Among patients with CAD, LM disease was not only a strong predictor of in-hospital mortality for PCI, but was also associated with high mortality, even after successful PCI for ACS (Wu et al., 2006; Puricel et al., 2011). These findings may explain why patients with an ST depression ECG pattern in our study had worse in-hospital and long-term clinical outcomes. Our study also confirms previous findings that advanced Killip score, old age, prior PTCA and advanced CKD are strong predictors of a long-term adverse outcome (Mueller et al., 2004; Charytan et al., 2009; Song et al., 2010; Rapsomaniki et al., 2014).

### Study limitations

Some limitations in this study should be noted. First, this was a retrospective cohort study. Second, this study did not include in the analysis NSTEMI patients who received conservative therapy. Therefore, this study might have selection bias. However, the clinical outcome was better in those patients who received in-hospital PCI compared with those patients who received conservative therapy, as reported in previous studies (Fox et al., 2006; Wong et al., 2009; Singh, 2005). All of our patients with significant CAD received PCI during hospitalization, and ST depression was still a strong predictor of long-term mortality and cardiac death.

## Conclusions

Our study findings suggest that among NSTEMI patients undergoing PCI, those presenting with an ST depression ECG pattern had a higher percentage of DM, advanced CKD and LM disease than patients without ST depression. Long-term outcomes, especially in terms of total mortality and cardiac death, were worse in patients with an ST depression ECG pattern than in patients without ST depression; this may be due to associated comorbidities.

##  Supplemental Information

10.7717/peerj.8796/supp-1 Data S1 Raw data

## Additional Information and Declarations

### Data Availability

The following information was supplied regarding data availability: The raw data is available in a Supplemental File.
